# Positive Association between *APOA5* rs662799 Polymorphism and Coronary Heart Disease: A Case-Control Study and Meta-Analysis

**DOI:** 10.1371/journal.pone.0135683

**Published:** 2015-08-26

**Authors:** Huadan Ye, Annan Zhou, Qiangxiao Hong, Linlin Tang, Xuting Xu, Yanfei Xin, Danjie Jiang, Dongjun Dai, Yirun Li, Dao Wen Wang, Shiwei Duan

**Affiliations:** 1 Zhejiang Provincial Key Laboratory of Pathophysiology, School of Medicine, Ningbo University, Ningbo, Zhejiang, China; 2 Center of Safety Evaluation, Zhejiang Academy of Medical Sciences, Hangzhou, Zhejiang, China; 3 Institute of Hypertension and Department of Internal Medicine, Tongji Hospital, Tongji Medical College, Huazhong University of Science and Technology, Wuhan, China; CSIR-INSTITUTE OF GENOMICS AND INTEGRATIVE BIOLOGY, INDIA

## Abstract

**Objective:**

Apolipoprotein A5 (*APOA5*) is associated with plasma triglyceride (TG) levels, a risk factor for coronary heart disease (CHD). This study explored the association between CHD and the *APOA5* rs662799 polymorphism.

**Methods:**

We collected 1,521 samples (783 CHD patients and 738 controls) for this case-control study. Meta-analysis was performed using Review Manager Software and Stata Software.

**Results:**

Significant differences were observed between CHD cases and controls at the level of both genotype (χ^2^ = 8.964, df = 2, P = 0.011) and allele (χ^2^ = 9.180, df = 1, P = 0.002, OR = 1.275, 95% CI = 1.089–1.492). A breakdown analysis by gender showed a significant association of *APOA5* rs662799 with CHD in males (χ^2^ = 7.770, df = 1, P = 0.005; OR = 1.331, 95% CI = 1.088–1.628). An additional meta-analysis using 21378 cases and 28428 controls established that rs662799 is significantly associated with CHD (P < 0.00001).

**Conclusion:**

Both our case-control study and meta-analysis confirm a significant association between *APOA5* rs662799 and CHD. In addition, our results suggest a male-specific association between the *APOA5* rs662799 polymorphism and CHD.

## Introduction

Coronary heart disease (CHD) is a type of cardiovascular disease that is caused by ischemia and hypoxia in the coronary artery [1] and is the leading cause of human deaths worldwide [2–4]. CHD is the most common cause of death among both men and women over the age of 50 [5]. Environmental factors associated with CHD include obesity, smoking, drinking, diabetes, arterial hypertension and dyslipidemia [6]. In addition, genetic factors are important for CHD [7].

*APOA5* is located in the apolipoprotein APOA1/C3/A4 gene cluster [8] on chromosome 11q23 [8,9]. *APOA5* is predominantly expressed in hepatocytes and secreted into the blood [10,11]. The APOA5 apolipoprotein plays a key role in the synthesis and removal of triglycerides (TG) [5]. Increased levels of apolipoprotein A5 are correlated with decreased TG levels in the serum [5].

Atherogenic dyslipidemia is a major risk factor for CHD [12–14], as are blood lipid levels [6]. Blood lipids mainly consist of low-density lipoprotein cholesterol (LDL-C), high-density lipoprotein cholesterol (HDL-C) and TG [12]. In addition to LDL-C and HDL-C levels, *APOA5* is associated with TG levels [13]. *APOA5* plays an important role in determining TG levels in serum [14]. TG interacts with lipoprotein lipase, an enzyme important for the central regulation of circulating TG levels [15]. In mice, over expression of *Apoa5* leads to decreased concentrations of TG in plasma, whereas a shortage of apoA5 causes hypertriglyceridemia, a risk factor for atherosclerosis and CHD [16]. These findings are consistent with observations in humans [17]. Taken together, these studies indicate that *APOA5* is associated with CHD [18–20].

*APOA5* rs662799 (-1131T>C) is a promoter polymorphism that was shown to be associated with increased levels of TG in young adult Indians [21]. In Italians, *APOA5* is associated with TG leaves and acute myocardial infarction (MI) [13]. The significant association of rs662799 with TG and CHD was validated in a Japanese population [22]. According to the HapMap database, there are ethnic differences in *APOA5* rs662799 (A>G). The minor allele frequency in European populations (HapMap-CEU) is 1.7%, much lower than the 13.3% observed in individuals of African descent (HapMap-YRI), 26.7% in Chinese (HapMap-CHB) and 28.9% in Japanese (HapMap-JPT). In our previous study, we could not detect a significant association between *APOA5* rs662799 and CHD [23], possibly due to a lack of power. Here, we increased the sample size to determine whether *APOA5* rs662799 plays a role in the risk of CHD in Han Chinese.

## Materials and Methods

### Sample collection

Samples from 1,521 unrelated individual inpatients were randomly collected from the Ningbo Lihuili Hospital and the Ningbo Yinzhou People's Hospital, Zhejiang, China. The samples included 783 cases of CHD (537 males and 246 females) and 738 controls (421 males and 317 females). All individuals were free from congenital heart disease, cardiomyopathy and severe liver or kidney disease. Details of the classified criteria have been described in our previous studies [2,24–27]. The study protocol was approved by the Ethical Committees of Ningbo Lihuili Hospital and Ningbo Yinzhou People's Hospital, and informed written consent was obtained from all subjects. The clinical and demographic details of CHD samples are summarized in S1 Table.

### SNP Genotyping

Genomic DNA was isolated from peripheral blood lymphocytes using a nucleic acid extraction automatic analyzer (Lab-Aid 820, Xiamen, China). PCR was performed on the ABI GeneAmp PCR System 9700 Dual 384-Well Sample Block Module (Applied Biosystems, Foster City, CA, USA). PCR conditions included an initial denaturation of 95°C for 2 min, followed by 45 cycles of 95°C for 30 sec, 56°C for 30 s, 72°C for 1 min and then a final extension at 72°C for 5 min. After purification by SAP Reaction, we proceeded with primer extension. The primer extension protocol included an initial denaturation at 94°C for 30 s, followed by 40 cycles of amplification (including 94°C for 5 s, 52°C for 5 s, 80°C for 5 s), 5 cycles of amplification (5 s at 52°C, 5 s at 80°C), a final extension at 72°C for 3 min after which samples were held at 4°C. Single nucleotide polymorphism genotyping was performed using the Sequenom Mass-ARRAY iPLEX platform per the manufacturer's instructions [28]. The primer sequences were 5’- ACGTTGGATGAGCATTTGGGCTTGCTCTCC-3’ (first primer), 5’-ACGTTGGATGTCTGAGCCCCAGGAACTGGA-3’ (second primer) and 5’- caGAACTGGAGCGAAAGT-3’ (extended primer).

### Publication retrieval and data extraction

**Fig 1:**
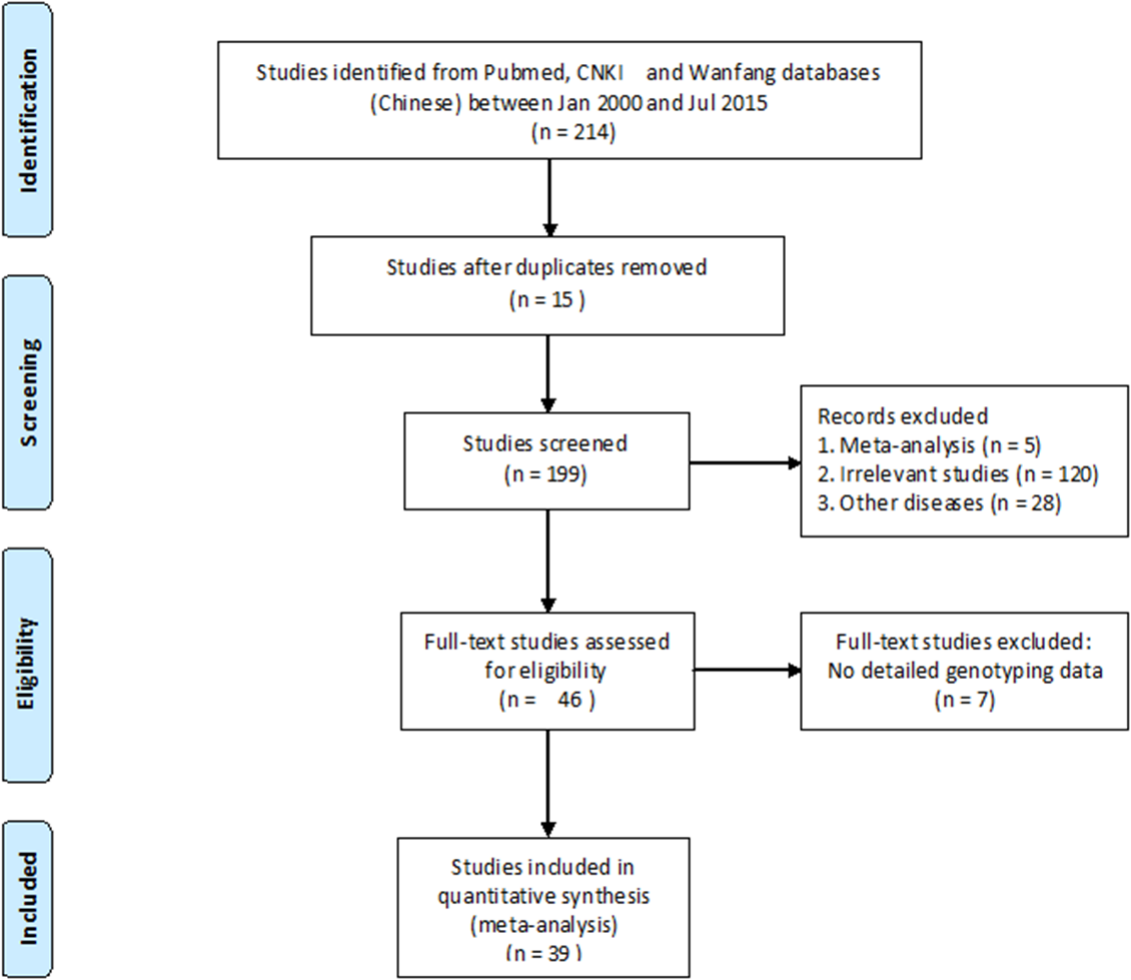
Flow chart of the meta-analysis.

The literatures were searched in the online databases including PubMed and Wanfang between Jan 2000 and Jul 2015. The keywords were “coronary heart disease”, “coronary artery disease” or “myocardial infarction” combined with “APOA5” and “rs662799” or “-1131T>C”. All of the case-control studies between APOA5 rs662799 and CHD were retrieved for the consideration of the current meta-analysis. All of the case-control studies between *APOA5* (rs662799) and CHD were considered to be eligible for the current meta-analysis. We only included studies that presented data on allele or genotype frequencies for both cases and controls and displayed a genotype distribution meeting Hardy-Weinberg equilibrium (HWE) [29]. Information in the meta-analysis included the first author’s name, publication year, country, ethnic group, number of alleles or genotypes and the total number of cases and controls. The details on the inclusion criteria included as follow:1) only the case-control studies on the association between rs662799 and CHD were included; 2) the eligible studies must contain the odds ratios (ORs) and 95% confidence intervals (CIs), or the genotype or allele information to calculate ORs and 95% Cis; 3) HWE should be met for the genotype distribution in the control group of the eligible studies if they have genotype information. We directly emailed the corresponding authors or called them (only for authors in China) for the missing information in their studies. There were 214 studies retrieved from the Wanfang and CNKI literature databases after searching for the keywords “coronary heart disease”, “coronary artery disease” or “myocardial infarction” combined with “*APOA5*” and “rs662799” or “-1131T>C”. After a series of selection procedures, we excluded 15 duplicate studies, 5 meta-analysis studies, 120 irrelevant studies, 28 studies on other diseases, and 7 studies without genotyping data (S1 File). In addition, we further downloaded the GWAS dataset from WTCCC research, then we imputed the information of rs662799 genotype by MaCH-Admix in WTCCC database, and added the data to the meta-analysis [30]. The remaining 40 case-control studies were qualified for our meta-analysis (Fig 1) [11,13,17,20,22,31–63].

### Statistical analyses

The HWE test was performed using the Arlequin program (version 3.5), and P > 0.05 was considered to be in HWE [64]. Genotype and allele distribution was compared between cases and controls by CLUMP22 software using 10,000 Monte Carlo simulations [65]. The odds ratio (OR) with a 95% confidence interval (CI) were determined using an online program, (http://faculty.vassar.edu/lowry/odds2x2.html) [23]. Meta-analysis was performed using the Review Manager software set to the fixed-effect or random-effect method (version 5.0, Cochrane Collaboration, Oxford, United Kingdom) [66]. Heterogeneity in the meta-analysis was assessed using the Q and I^2^ tests. An I^2^ > 50% indicated the existence of heterogeneity among the studies in the meta-analysis. Publication bias was shown by Begg’s funnel plot analysis, which was generated with Stata software (version 11.0, Stata Corporation, College Station, TX, USA). P values < 0.05 were significant.

## Results

No departure from HWE was observed for the *APOA5* rs662799 polymorphism in cases (P = 0.220) or controls (P = 0.544). Genotypic and allelic comparisons between cases and controls are shown in Table 1. Our data show that rs622799 is associated with the risk of CHD (genotype: χ^2^ = 8.964, df = 2, P = 0.011; allele: P = 0.002; OR = 1.275, 95% CI = 1.089–1.492). A further gender-stratified association shows that rs662799 is significantly associated with CHD in males (Table 1, genotype: χ^2^ = 7.486, df = 2, P = 0.024; allele: χ^2^ = 7.770, df = 1, P = 0.005) but not in females. In addition, frequency of the rs662799-G allele is significantly higher in male cases (31.5%) than in male controls (25.7%, P = 0.005; OR = 1.331, 95% CI = 1.088–1.628; Table 1). A further breakdown analysis by age shows that the frequency of rs662799-G is significantly higher in CHD cases with ages ranging from 55 to 65 years (31.5% versus 25.0%, χ^2^ = 5.700, df = 1, P = 0.017, OR = 1.383, 95% CI = 1.059–1.805; Table 2).

**Table 1:** Genotype and allele frequencies in cases and controls.

		Genotype [n, (%)]			χ^2^	P (df = 2)	HWE	Allele (counts)		χ^2^	P (df = 1)	OR (95% CI)
APOA5	Rs662799	GG	AG	AA				G	A			
All	Cases (N = 783)	85(10.9%)	323(41.3%)	375(47.8%)			0.220	493	1,073			
	Controls (N = 738)	55(7.5%)	281(38.1%)	402(54.4%)	8.964	0.011	0.544	391	1,085	9.180	0.002	1.275 (1.089–1.492)
Male	Cases (N = 537)	58(10.8%)	222(41.3%)	257(47.9%)			0.335	338	736			
	Controls (N = 421)	32(7.6%)	152(36.1%)	237(56.3%)	7.486	0.024	0.272	216	626	7.770	0.005	1.331 (1.088–1.628)
Female	Cases (N = 246)	27(11.0%)	101(41.1%)	118(47.9%)			0.445	155	337			
	Controls (N = 317)	23(7.3%)	129(40.7%)	165(52.0%)	2.622	0.270	0.746	175	459	2.040	0.153	1.206 (0.932–1.561)

**Table 2:** Genotype and allele frequencies in cases and controls with different age ranges.

		Genotype [n, (%)]			χ^2^	P (df = 2)	HWE	Allele (counts)		χ^2^	P (df = 1)	OR (95% CI)
Age	Rs662799	GG	GA	AA				G	A			
55≤	Cases (N = 179)	20(11.2%)	68(38.0%)	91(50.8%)			0.188	108	250			
	Controls (N = 239)	18(7.5%)	93(38.9%)	128(53.6%)	1.660	0.436	0.846	129	349	1.02	0.313	1.169 (0.863–1.582)
55–65	Cases (N = 271)	27(10.0%)	117(43.2%)	127(46.8%)			0.994	171	371			
	Controls (N = 268)	17(6.3%)	100(37.3%)	151(56.4%)	5.660	0.059	0.935	134	402	5.700	0.017	1.383 (1.059–1.805)
≥65	Cases (N = 333)	38(11.4%)	138(41.4%)	157(47.2%)			0.363	214	452			
	Controls (N = 231)	20(8.7%)	88(38.1%)	123(53.2%)	2.409	0.300	0.456	128	334	2.530	0.112	1.235 (0.952–1.603)

### Meta-analysis

**Fig 2:**
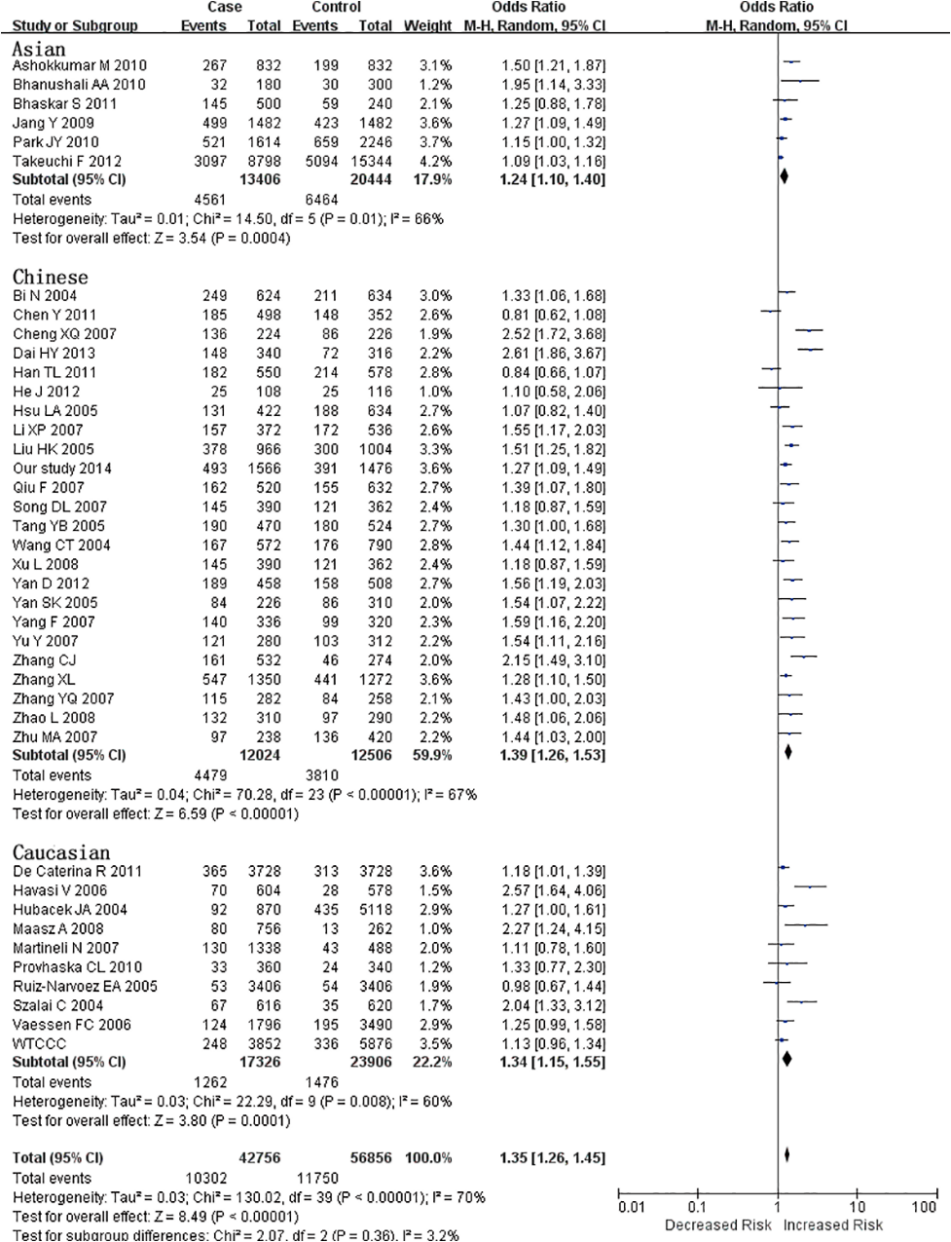
Forest plots of APOA5 rs662799 polymorphism with CHD risk in the Chinese, the Caucasian and the Asian populations*. *Events, the number of G alleles; total, total number of A and G alleles; our study: the CHD cases and controls in our study.

**Fig 3:**
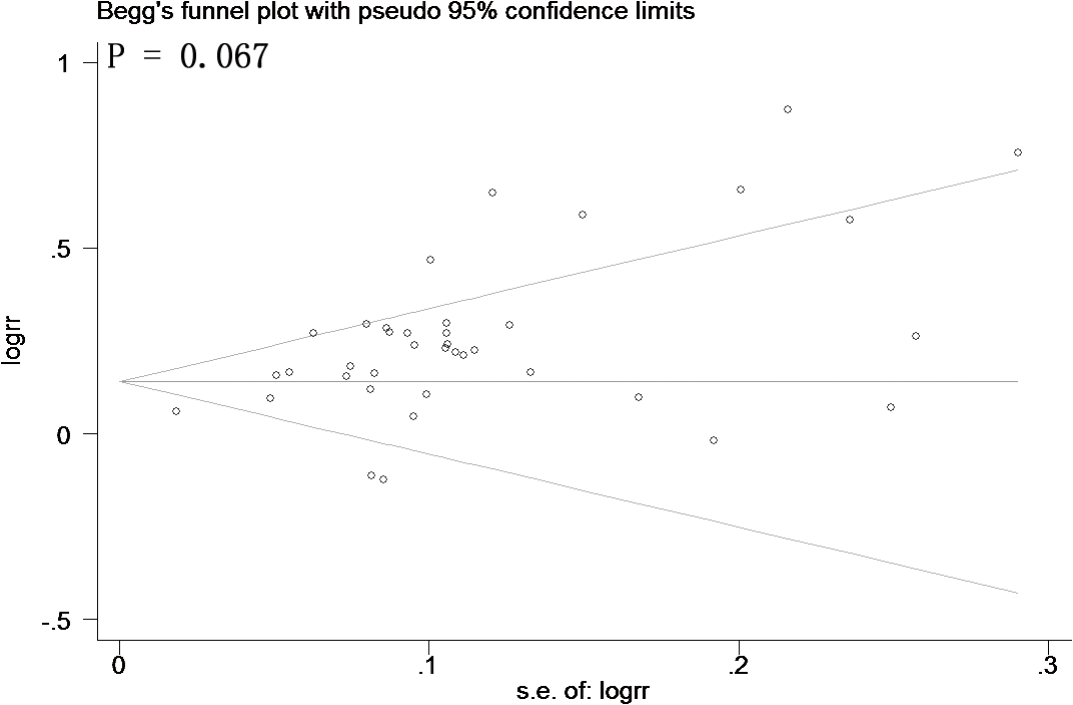
Begg’s funnel plot for the association between APOA5 rs662799 and CHD*. *Horizontal axis represents the standard error of log rr. Vertical axis represents the log rr. The s.e. denotes standard error.

**Fig 4:**
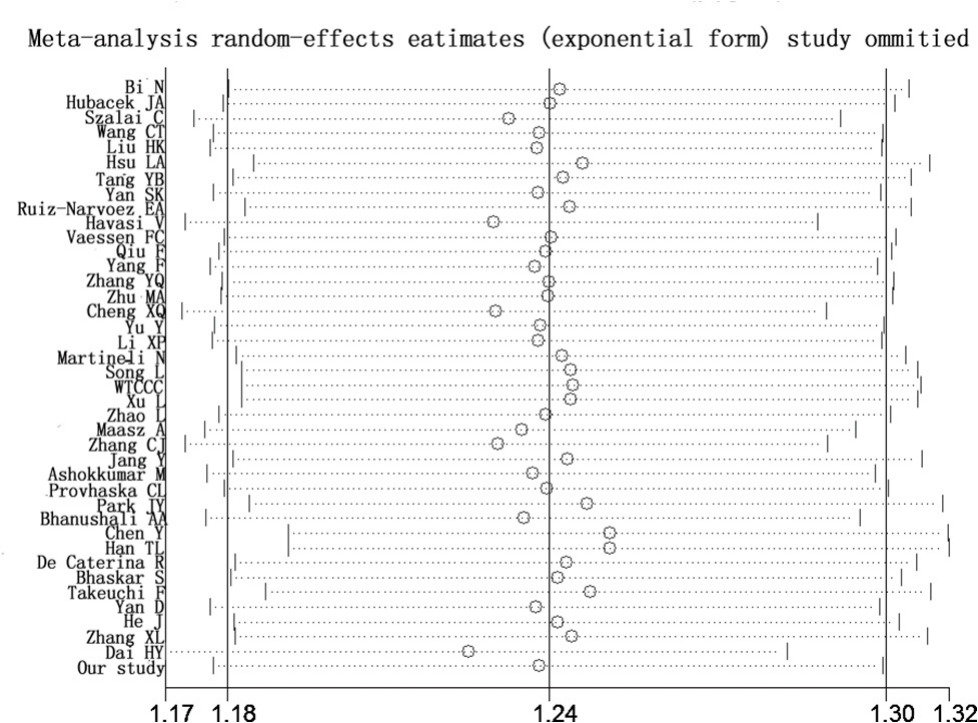
Sensitivity analysis for the APOA5 rs662799 polymorphism with CHD.

Searching the existing literature databases, we found 40 case-control studies, 30 more cases than were used in the most recently published meta-analysis in 2013 [23]. Therefore, we performed an updated meta-analysis to investigate the link between rs662799 and CHD. Information from these 40 eligible studies and our case-control study are shown in Table 3. Among the 40 eligible studies in the current meta-analysis, 7 studies only had allelic information. Therefore, allele-based model was applied in the meta-analysis. For the meta-analysis with moderate heterogeneity (I^2^ < 50%), we selected a fixed-effect model for the meta-analysis, otherwise, the random-effect model was used for the meta-analysis with great heterogeneity (I^2^ > = 50%). The current meta-analysis has great heterogeneity (I^2^ = 70%), therefore random-effect model was used. As shown in Fig 2, subgroup meta-analysis by major ethnic groups also indicates a significant association between *APOA5* rs662799 and CHD in Asians (P = 0.01, I^2^ = 66%), Chinese (P < 0.000001, I^2^ = 67%) and Caucasians (P = 0.008, I^2^ = 60%). The meta-analyses show no publication bias by Begg’s funnel plot analysis (Fig 3). Furthermore, sensitivity analysis suggests that the conclusion is not biased by any individual study (Fig 4).

**Table 3:** Detailed information of the *APOA5* rs662799 cases included in the meta-analysis.

Year	Author	Ethnic Group	NO. case/controls	NO. A allele	NO. G allele
2004	Bi N [40]	Chinese	312/317	375/423	249/211
2004	Hubacek JA [41]	Caucasian	435/2,559	778/4,683	92/435
2004	Szalai C [42]	Hungarian	308/310	549/585	67/35
2004	Wang CT [33]	Chinese	286/395	405/614	167/176
2005	Liu HK [43]	Chinese	483/502	588/704	378/300
2005	Hsu LA [44]	Chinese	211/317	291/446	131/188
2005	Tang YB [45]	Chinese	235/262	280/344	190/180
2005	Yan SK [46]	Chinese	113/155	142/224	84/86
2005	Ruiz-Narvoez EA [47]	Costa Rican	1,703/1,703	3,353/3,352	53/54
2006	Havasi V [48]	Hungarian	302/289	534/550	70/28
2006	Vaessen FC [49]	British	898/1,745	1,672/3,295	124/195
2007	Qiu F [57]	Chinese	260/316	358/477	162/155
2007	Yang F [31]	Chinese	168/160	196/221	140/99
2007	Zhang YQ [58]	Chinese	141/129	167/174	115/84
2007	Zhu MA [39]	Chinese	119/210	141/284	97/136
2007	Cheng XQ [37]	Chinese	112/113	138/162	136/86
2007	Yu Y [50]	Chinese	140/156	159/209	121/103
2007	Li XP[38]	Chinese	186/268	215/364	157/172
2007	Martineli N [51]	Italian	669/244	1,208/445	130/43
2007	Song DL [29]	Chinese	195/181	245/241	145/121
2007	WTCCC [30]	Caucasian	1926/2938	3604/5540	248/336
2008	Xu L [59]	Chinese	195/181	245/241	145/121
2008	Zhao L [60]	Chinese	155/145	178/193	132/97
2008	Maasz A [52]	Hungarian	378/131	676/249	80/13
2009	Zhang CJ [35]	Chinese	266/137	371/226	161/46
2009	Jang Y [17]	Korean	741/741	983/1,059	499/423
2010	Ashokkumar M [53]	Indian	416/416	565/633	267/199
2010	Provhaska CL [54]	Brazilian	180/170	327/316	33/24
2010	Park JY [11]	Korean	807/1,123	1,093/1,587	521/659
2010	Bhanushali AA [55]	Indian	90/150	58/120	32/30
2011	Chen Y [61]	Chinese	249/176	313/204	185/148
2011	Han TL [32]	Chinese	275/289	368/364	182/214
2011	Raffaele De Caterina [13]	Italian	1,864/1,864	3,319/3,423	365/313
2011	Bhaskar S [56]	Indian	250/120	355/181	145/59
2012	Takeuchi F [22]	Japanese	4,399/7,672	5,701/10,250	3,097/5,094
2012	Yan D [20]	Chinese	229/254	269/350	189/158
2012	He J [30]	Chinese	54/58	83/91	25/25
2012	Zhang XL [36]	Chinese	675/636	803/831	547/441
2013	Dai HY [34]	Chinese	158/130	244/138	72/122
2014	Our study	Chinese	783/738	1,073/1,085	493/391

## Discussion

Our results show that the rs662799 polymorphism in the *APOA5* gene is significantly associated with CHD in Han Chinese (P = 0.011). The minor G allele of *APOA5* rs662799 may increase the risk of CHD by 27.5% (P = 0.002, OR = 1.275, 95% CI = 1.089–1.492). Consistent with previous reports, the rs662799-G allele is associated with higher leaves of TG in both CHD patients and controls [13,67]. A power calculation for *APOA5* rs662799 indicates that our study has 85.9% power to detect significance in the association test.

Environmental factors, such as gender and age, are important factors of CHD. The prevalence of CHD in females was different from males [2,68,69]. Evidence has shown that patients older than 65 years have a higher cardiovascular morbidity and mortality [70,71]. In the current meta-analysis, we were unable to perform the subgroup meta-analysis by the age or gender due to a paucity of related information in the involved studies.

Gender and age are independence risk of CHD [4,72–74]. Epidemiologic evidence suggests that the risk of morbidity and mortality are higher in male CHD patients than in females [75]. Our data show a strong association between *APOA5* rs662799 and CHD in the male group, providing a novel molecular explanation for the gender disparity observed in CHD. In addition, we showed a statistically significant difference between rs662799 and CHD in the subgroup aged from 55–65, although the underlying mechanism will require additional studies.

The frequency of the *APOA5* rs662799 polymorphism varies greatly among different populations. The rs662799-G allele frequency is 26.7% in Chinese populations, similar to that in Japanese populations (29.1%). However, the Chinese frequency is much higher than that in European populations (1.7%). Nevertheless, accumulating evidence indicates a strong association between *APOA5* rs662799 and CHD among different populations. In addition to *APOA5* rs662799, there are associations between other *APOA5/A4/C3/A1* polymorphisms and CHD, which include *APOA5* rs3135506 and *APOA/A4/C3/A1* cluster haplotypes [76]. Further functional analysis is needed to discriminate the relationship among these polymorphisms.

There were other seven *APOA5* polymorphisms involved in the genetic studies (S2 Table). However, rs3135506 (n = 7) [43,45,49,53,56,76,77] and -12238T/C (n = 1)[78] were tested for the association of CHD. Thus, we only included rs662799 in the current meta-analysis. Among the published GWAS related to the current meta-analysis, we didn’t find any direct information that could be applied in the current meta-analysis [12,67,79]. We further and added the WTCCC data to the meta-analysis. Please see the following figure for the updates (Fig 2). The current meta-analysis includes 40 studies comprised of 21378 cases and 28428 controls from 10 ethnic populations. Our meta-analysis contains at least 26 case studies and 3 ethnic populations more than were included in the last five meta-analyses published [23, 80–83]. All of the meta-analyses indicate that the *APOA5* rs662799 polymorphisms associated with CHD in the Chinese population, although many of the studies did not include a subgroup analysis stratified by ethnicity.

Despite the merits of our meta-analysis, there are limitations that must be considered. Our meta-analysis only includes studies from Asian and Caucasian populations. Therefore, it might not be an accurate representation of other ethnicities, such as African populations. Publication and language bias might exist in the case control studies [84]. The current meta-analysis was involved with 10 Caucasian and 30 Asian studies. Among the Asian studies, there were 24 Chinese studies (7 in English and 17 in Chinese). A further check for the minor allele frequency report in the HapMap International Project, we found the MAF in Europeans was 1.7% which was much less than 26.7% in Chinese and 29.1% in Japanese. However, subgroup meta-analyses by ethnicity found significant association of *APOA5* rs662799 and CHD in both Europeans and Asians. There may also be a selection bias in our meta-analysis, which only included studies published in English or Chinese. Finally, standards for diagnosis may vary due to differences in the inclusion of CHD cases and non-CHD controls.

In summary, our case-control and meta-analysis demonstrates that the frequency of the *APOA5* rs662799-G allele is significantly increased in CHD cases compared with controls. Furthermore, *APOA5* rs662799 interacts with both gender and age in the association with CHD.

## Supporting Information

S1 PRISMA Checklist PRISMA Checklist. (DOC)

S1 File Supplemental document 1: The excluded 7 studies without genotyping data. (DOCX)

S1 Table The clinical and demographic details of CHD and non-CHD samples*. * p values were determined by the Wilcoxon-Mann-Whitney test.
(DOCX)

S2 Table Other seven *APOA5* polymorphisms involved in the genetic studies. (DOC)
