# Supplementary material for: Positive Association between APOA5 rs662799 Polymorphism and Coronary Heart Disease: A Case-Control Study and Meta-Analysis
Source: PLoS One. 2015 Aug 26;10(8):e0135683. doi: 10.1371/journal.pone.0135683 (PMC4550406; doi:10.1371/journal.pone.0135683)
Supplement: S1 PRISMA Checklist — (DOC) [file pone.0135683.s001.doc]

| **Section/topic** | **#** | **Checklist item** | **Reported on page #** |
| --- | --- | --- | --- |
| **TITLE** | | |  |
| Title | 1 | Positive association between APOA5 rs662799 polymorphism and coronary heart disease: a case-control study and an updated meta-analysis | Title |
| **ABSTRACT** | | |  |
| Structured summary | 2 | Objective: Apolipoprotein A5 (APOA5) was shown to be associated with the level of plasma triglyceride (TG), a risk factor of coronary heart disease (CHD). The aim of present study was to explore the association between APOA5 rs662799 polymorphism and CHD.  Methods: A total of 1521 samples (783 CHD patients and 738 controls) were collected in the case-control study. Meta-analysis was performed using Review Manager Software and Stata Software.  Results: Significant differences were observed between CHD cases and controls on the levels of both genotype (χ² = 8.964, df = 2, P = 0.011) and allele (χ² = 9.180, df = 1, P = 0.002, OR = 1.275, 95% CI = 1.089 - 1.492). A breakdown analysis by gender showed a significant association of APOA5 rs662799 with CHD in males (χ² = 7.770, df = 1, P = 0.005; OR = 1.331, 95% CI = 1.088 - 1.628). A further updated meta-analysis among 21378 cases and 28428 controls established that rs662799 was significantly associated with CHD (P < 0.00001).  Conclusion: Both our case-control study and meta-analysis have confirmed the significant association of APOA5 rs662799 with CHD. In addition, our results suggested a male-specific association between APOA5 rs662799 polymorphism and CHD. | 2 |
| **INTRODUCTION** | | |  |
| Rationale | 3 | Coronary heart disease (CHD) is caused by ischemia and hypoxia in the coronary artery. CHD is a type of cardiovascular disease that has become the top leading cause of human deaths in the world. CHD is considered to be the most principal cause of death among both men and women over the 50 years old. The environmental factors of CHD include obesity, smoking, drinking, diabetes, arterial hypertension and dyslipidemia. In addition, genetic factors are also important for CHD.  APOA5 is located in the apolipoprotein APOA1/C3/A4 gene cluster on chromosome 11q23. APOA5 was predominantly expressed in hepatocytes and Secreted into the blood. APOA5 apolipoprotein plays a key role in the [synthesis](app:ds:synthetic) and removal of triglyceride (TG). Increased A5 apolipoprotein correlates with decreased TG level in the serum.  Evidences had shown that atherogenic dyslipidemia was a major risk factor of CHD. Blood lipids levels were related to the risk of CHD. The blood lipids mainly consisted of low-density lipoprotein cholesterol (LDL-C), high-density lipoprotein cholesterol (HDL-C) and TG. APOA5 was associated with TG level as well as LDL-C and HDL-C levels. APOA5 played an important role in determining TG level in the serum, which interacted with lipoprotein lipase, an enzyme participating in the central regulation of circulating TG levels. In mice, the over expression of Apoa5 led to decreased plasma TG concentration, whereas the shortage of apoA5 resulted in hypertriglyceridemia, a risk factor for atherosclerosis and CHD. These findings were also observed in humans. The above evidence indicated that APOA5 was associated with CHD.  APOA5 rs662799 (-1131T>C) is a promoter polymorphism that was shown to be associated with raised TG level in Indian young adulthood. APOA5 rs662799 was also shown to be associated with TG level in Italian samples and was associated with acute myocardial infarction (MI). This significant association of rs662799 with TG and CHD was later validated in Japanese population. According to the HapMap database, ethnic difference existed for APOA5 rs662799 (A>G). The minor allele frequency in European population (HapMap-CEU) is 1.7%, which was much lower than 13.3% in African descents (HapMap-YRI), 26.7% in Chinese (HapMap-CHB) and 28.9% in Japanese (HapMap-JPT). | 3-4 |
| Objectives | 4 | Our previous study was unable to find a significant association between APOA5 rs662799 and CHD that might be due to a lack of power. Here, we expanded the sample size to establish the role of APOA5 rs662799 in the risk of CHD in Han Chinese. | 4 |
| **METHODS** | | |  |
| Protocol and registration | 5 | No. |  |
| Eligibility criteria | 6 | All the case-control studies between APOA5 (rs662799) and CHD were considered to be eligible for the current meta-analysis. And the included studies should contain allele or genotype frequencies in both cases and controls, and the genotype distribution should meet Hardy-Weinberg equilibrium (HWE) | 6 |
| Information sources | 7 | In the current meta-analysis, we collected 39 studies from the literature databases of PubMed, Wanfang and China National Knowledge Infrastructure (CNKI) Jan 2000 and Jul 2015. | 6 |
| Search | 8 | The keywords were “coronary heart disease”, “coronary artery disease” or “myocardial infarction” combined with “APOA5” and “rs662799” or “-1131T>C”. All of the case-control studies between APOA5 rs662799 and CHD were retrieved for the consideration of the current meta-analysis. | 6 |
| Study selection | 9 | The retrieved information in the meta-analysis included the first author’s name, publication year, country, ethnic group, number of alleles or genotypes, and total number of cases and controls. The details on the inclusion criteria included as follow:1) only the case-control studies on the association between rs662799 and CHD were included; 2) the eligible studies must contain the odds ratios (ORs) and 95% confidence intervals (CIs), or the genotype or allele information to calculate ORs and 95% Cis; 3) HWE should be met for the genotype distribution in the control group of the eligible studies if they have genotype information. | 6 |
| Data collection process | 10 | There were 214 studies retrieved from the Wanfang and China National Knowledge Infrastructure (CNKI) literature database after searching the keywords of “coronary heart disease” or “coronary artery disease” or “myocardial infarction” combined with “APOA5” and “rs662799” or “-1131T>C”. After a series of selection procedure, we excluded 15 duplicate studies, 5 meta-analysis studies, 120 irrelevant studies, 28 other disease studies, and 7 studies without genotyping data. Finally, 39 case-control studies were qualified for our meta-analysis (Figure 1) | 6, Fig1 |
| Data items | 11 | The current meta-analysis is involved with 40 studies among 21378 cases and 28428 controls from 10 ethnic populations. | 10 |
| Risk of bias in individual studies | 12 | Heterogeneity in the meta-analysis was assessed using the Q and I2 tests. An I2 > 50% showed there exist heterogeneity among the studies in the meta-analysis. Publication bias was shown using the Begg’s funnel plot analysis by Stata software (version 11.0, Stata Corporation, College Station, TX). | 6-7 |
| Summary measures | 13 | Review manager 5 and stata 11.0 were used for meta-analysis. Random-effect Mantel-Haenszel ORs and their 95% CIs were computed for the polymorphism to evaluate the contribution of rs662799 to the risk of coronary heart disease. | 6-7 |
| Synthesis of results | 14 | For the 40 studies, we evaluated overall ORs in case-control studies from coronary heart disease subjects. | 6-7 |

Page 1 of 2

| **Section/topic** | **#** | **Checklist item** | **Reported on page #** |
| --- | --- | --- | --- |
| Risk of bias across studies | 15 | Sensitivity analysis suggested that the conclusion was not biased by any individual study | 8 |
| Additional analyses | 16 | No. |  |
| **RESULTS** | | |  |
| Study selection | 17 | A search in the existing literature databases found there were a total of 40 existing case-control studies, 31 more than the latest meta-analysis in 2013 | 8 |
| Study characteristics | 18 | The current meta-analysis is involved with 40 studies among 21378 cases and 28428 controls from 10 ethnic populations. | 10 |
| Risk of bias within studies | 19 | The meta-analyses showed no publication bias by Begg’s funnel plot analysis (Figure3) | 8 |
| Results of individual studies | 20 | The retrieved information of 39 eligible studies and our case-control study was shown in Table 3 | 8 |
| Synthesis of results | 21 | Our results showed a significant association between APOA5 rs662799 and CHD (P < 0.00001; Figure 2) under random model in the meta-analysis. | 8 |
| Risk of bias across studies | 22 | There were no difference among Chinese, Asian and Caucasian subgroups | Fig 2 |
| Additional analysis | 23 | Furthermore, sensitivity analysis suggested that the conclusion was not biased by any individual study (Figure 4). | 8 |
| **DISCUSSION** | | |  |
| Summary of evidence | 24 | Our results show that the rs662799 polymorphism in the APOA5 gene is significantly associated with CHD in Han Chinese (P = 0.011). The minor G allele of APOA5 rs662799 may increase the risk of CHD by 27.5% (P = 0.002, OR = 1.275, 95% CI = 1.089 - 1.492). Consistent with previous reports, the rs662799-G allele is associated with higher leaves of TG in both CHD patients and controls. A power calculation for APOA5 rs662799 indicates that our study has 85.9% power to detect significance in the association test.  Environmental factors, such as gender and age, are important factors of CHD. The prevalence of CHD in females was different from males. Evidence has shown that patients older than 65 years have a higher cardiovascular morbidity and mortality. In the current meta-analysis, we were unable to perform the subgroup meta-analysis by the age or gender due to a paucity of related information in the involved studies.  Gender and age are independence risk of CHD. Epidemiologic evidence suggests that the risk of morbidity and mortality are higher in male CHD patients than in females. Our data show a strong association between APOA5 rs662799 and CHD in the male group, providing a novel molecular explanation for the gender disparity observed in CHD. In addition, we showed a statistically significant difference between rs662799 and CHD in the subgroup aged from 55-65, although the underlying mechanism will require additional studies.  The frequency of the APOA5 rs662799 polymorphism varies greatly among different populations. The rs662799-G allele frequency is 26.7% in Chinese populations, similar to that in Japanese populations (29.1%). However, the Chinese frequency is much higher than that in European populations (1.7%). Nevertheless, accumulating evidence indicates a strong association between APOA5 rs662799 and CHD among different populations. In addition to APOA5 rs662799, there are associations between other APOA5/A4/C3/A1 polymorphisms and CHD, which include APOA5 rs3135506 and APOA/A4/C3/A1 cluster haplotypes. Further functional analysis is needed to discriminate the relationship among these polymorphisms.  There were other seven APOA5 polymorphisms involved in the genetic studies (Table S2). However, rs3135506 (n = 7) and -12238T/C (n = 1) were tested for the association of CHD. Thus, we only included rs662799 in the current meta-analysis. Among the published GWAS related to the current meta-analysis, we didn’t find any direct information that could be applied in the current meta-analysis. We further and added the WTCCC data to the meta-analysis. Please see the following figure for the updates (Figure 2). The current meta-analysis includes 40 studies comprised of 21378 cases and 28428 controls from 10 ethnic populations. Our meta-analysis contains at least 26 case studies and 3 ethnic populations more than were included in the last five meta-analyses published. All of the meta-analyses indicate that the APOA5 rs662799 polymorphisms associated with CHD in the Chinese population, although many of the studies did not include a subgroup analysis stratified by ethnicity. | 10-11 |
| Limitations | 25 | Despite the merits of our meta-analysis, there are limitations that must be considered. Our meta-analysis only includes studies from Asian and Caucasian populations. Therefore, it might not be an accurate representation of other ethnicities, such as African populations. Publication and language bias might exist in the case control studies. The current meta-analysis was involved with 10 Caucasian and 30 Asian studies. Among the Asian studies, there were 24 Chinese studies (7 in English and 17 in Chinese). A further check for the minor allele frequency report in the HapMap International Project, we found the MAF in Europeans was 1.7% which was much less than 26.7% in Chinese and 29.1% in Japanese. However, subgroup meta-analyses by ethnicity found significant association of APOA5 rs662799 and CHD in both Europeans and Asians. There may also be a selection bias in our meta-analysis, which only included studies published in English or Chinese. Finally, standards for diagnosis may vary due to differences in the inclusion of CHD cases and non-CHD controls. | 11 |
| Conclusions | 26 | In summary, our case-control and meta-analysis demonstrates that the frequency of the APOA5 rs662799-G allele is significantly increased in CHD cases compared with controls. Furthermore, APOA5 rs662799 interacts with both gender and age in the association with CHD. | 12 |
| **FUNDING** | | |  |
| Funding | 27 | This work was supported by grants from: National Natural Science Foundation of China (31100919 and 81371469), Natural Science Foundation of Zhejiang Province (LR13H020003), the K. C. Wong Magna Fund in Ningbo University, Zhejiang Provincial Bureau of Traditional Chinese Medicine (2013ZZ003) and the Sciences Technology Department of Zhejiang Province (2013F20005). | 14 |

*From:*  Moher D, Liberati A, Tetzlaff J, Altman DG, The PRISMA Group (2009). Preferred Reporting Items for Systematic Reviews and Meta-Analyses: The PRISMA Statement. PLoS Med 6(6): e1000097. doi:10.1371/journal.pmed1000097

For more information, visit: **www.prisma-statement.org**.

Page 2 of 2
