# Supplementary material for: Positive Association between APOA5 rs662799 Polymorphism and Coronary Heart Disease: A Case-Control Study and Meta-Analysis
Source: PLoS One. 2015 Aug 26;10(8):e0135683. doi: 10.1371/journal.pone.0135683 (PMC4550406; doi:10.1371/journal.pone.0135683)
Supplement: S1 File — (DOCX) [file pone.0135683.s002.docx]

The excluded 7 studies without genotyping data were listed in below:

1. Lee KW, Ayyobi AF, Frohlich JJ, Hill JS. APOA5 gene polymorphism modulates levels of triglyceride, HDL cholesterol and FERHDL but is not a risk factor for coronary artery disease. Atherosclerosis, 2004, 176(1):165-72.
2. Talmud PJ, Martin S, Taskinen MR, Frick MH, Nieminen MS, et al. APOA5 gene variants, lipoprotein particle distribution, and progression of coronary heart disease: results from the LOCAT study. J Lipid Res, 2004, 45(4):750-6.
3. Lai CQ, Demissie S, Cupples LA, Zhu Y, Adiconis X, et al. Influence of the APOA5 locus on plasma triglyceride, lipoprotein subclasses, and CVD risk in the Framingham Heart Study. J Lipid Res, 2004, 45(4):750-6. 45(11):2096-105.
4. Triglyceride Coronary Disease Genetics Consortium and Emerging Risk Factors Collaboration, Triglyceride-mediated pathways and coronary disease: collaborative analysis of 101 studies. Lancet. 2010, 375(9726):1634-9.
5. Wei HX, Zhang K, Qiu F, Ni J, Li L et al. The relationship between serum apolipoprote in A5 and coronary heart disease. Chin J Clin Lab Sci. 2010, 28(4): 276-8.
6. Evans D, Buchwald A, Beil FU. The single nucleotide polymorphism -1131T>C in the apolipoprotein A5 (APOA5) gene is associated with elevated triglycerides in patients with hyperlipidemia. J Mol Med. 2003, 81(10):645-54.
7. Saleheen D, Soranzo N, Rasheed A, Scharnagl H, Gwilliam R. Genetic Determinants of Major Blood Lipids in Pakistanis Compared With Europeans. Circ Cardiovasc Genet. 2010, 3(4):348-57.
