# Supplementary material for: Positive Association between APOA5 rs662799 Polymorphism and Coronary Heart Disease: A Case-Control Study and Meta-Analysis
Source: PLoS One. 2015 Aug 26;10(8):e0135683. doi: 10.1371/journal.pone.0135683 (PMC4550406; doi:10.1371/journal.pone.0135683)
Supplement: S1 Table — * p values were determined by the Wilcoxon-Mann-Whitney test. (DOCX) [file pone.0135683.s003.docx]

**S1 Table: The clinical and demographic details of CHD and non-CHD samples.**

| **Characteristics** | **Case** | **Control** | **P*** |
| --- | --- | --- | --- |
| LDL-C (mmol/L) | 2.58±0.94 | 2.57±0.85 | 0.76 |
| Total cholesterol (mmol/L) | 4.36±1.14 | 4.40±1.03 | 0.37 |
| HDL-C (mmol/L) | 1.23±4.23 | 1.16±0.30 | 8.78E-6 |
| Triglyceride (mmol/L) | 1.71±1.34 | 1.59±0.96 | 0.19 |
| ApoA I (g/L) | 1.01±0.34 | 1.05±0.23 | 2.48E-6 |
| ApoB (g/L) | 0.79±0.28 | 0.83±0.48 | 0.09 |
| ApoE (g/L) | 4.51±3.04 | 4.86±9.69 | 0.34 |
| Lipoprotein (a) (mg/dL) | 41.71±123.79 | 76.80±150.12 | 7.35E-4 |
| C-reactionprotein (mg/L) | 7.35±15.19 | 5.93±15.82 | 0.03 |
| Albumin (g/L) | 40.91±4.32 | 41.33±3.91 | 0.03 |
| Globulin (g/L) | 25.11±4.18 | 24.77±4.55 | 0.33 |
| A/G | 1.70±0.82 | 1.76±0.99 | 0.06 |
| Alanine aminotransferase (U/L) | 27.35±22.91 | 29.63±38.20 | 0.64 |
| Aspartate transaminase (U/L) | 37.42±60.38 | 34.13±52.92 | 0.23 |
| Alkaline phosphatase (U/L) | 71.61±24.61 | 68.36±22.46 | 0.04 |
| Glutamyl transpeptidase (U/L) | 38.96±47.14 | 37.26±62.60 | 0.02 |

*: p values were determined by the Wilcoxon-Mann-Whitney test.
