# Supplementary material for: Positive Association between APOA5 rs662799 Polymorphism and Coronary Heart Disease: A Case-Control Study and Meta-Analysis
Source: PLoS One. 2015 Aug 26;10(8):e0135683. doi: 10.1371/journal.pone.0135683 (PMC4550406; doi:10.1371/journal.pone.0135683)
Supplement: S2 Table — (DOC) [file pone.0135683.s004.doc]

**S2 Table:** Other seven *APOA5* polymorphisms involved in the genetic studies

| SNP | Number of studies | Topic |
| --- | --- | --- |
| rs2266788 | 3 | familial combined hyperlipidemia (n =1);  lipid (n =2) |
| rs651821 | 5 | familial combined hyperlipidemia (n =1);  lipid (n =4) |
| rs3135506 | 23 | CHD (n = 7);  familial combined hyperlipidemia (n =1);  diabetes (n =2);  lipid (n =17) |
| -12238T/C | 1 | CHD |
| 457G>A | 1 | lipid |
| 1259T>C | 3 | lipid |
| IVS3+ 476G>A | 2 | lipid |
